# Improved stability of steerable sheath access by femoro-femoral crossover wire in branched stent graft repair of complex thoraco-abdominal aortic aneurysms

**DOI:** 10.1515/iss-2020-0006

**Published:** 2020-09-07

**Authors:** Ingolf Töpel, Thomas Betz, Markus Steinbauer, Christian Uhl

**Affiliations:** Department of Vascular Surgery, KH Barmherzige Brüder Regensburg, Regensburg, Germany

**Keywords:** aortic aneurysm, branched endograft, complex endovascular repair, retrograde access, steerable sheath

## Abstract

**Purpose:**

The purpose of this study was to describe a technique to catheterize antegrade branches of a branched aortic endograft by using a steerable sheath stabilized by a through-and-through wire via a femoral access.

**Technique:**

After implantation of a branched endovascular graft, a steerable 8.5 F sheath is advanced from the femoral access. After placing the sheath proximal to the branches, a 0.014″ through-and-through wire is established to the contralateral femoral access which is held under slight traction after the curved tip of the sheath is brought into the 180° position. Then catheterization, wire exchange and deployment of the bridging stent is done in standard fashion.

**Conclusion:**

The use of a through-and-through wire with a steerable sheath for retrograde femoral access adds stability and precision to this technique. It has the potential to reduce the risk of preoperative stroke in complex aortic endovascular repair by avoiding upper extremity access.

## Introduction

Endovascular repair of thoraco-abdominal aortic aneurysms frequently involves the use of downward facing, antegrade branches. Upper extremity access (brachial, axillary or subclavian artery) has been used regularly for catheterization of these branches and the target vessels and has been proven to be save and feasible [1]. One of the major drawbacks of placing large diameter sheaths across the arch is the risk of perioperative stroke [2]. In some cases, arterial access from the arm can be challenging and dangerous due to low arterial diameter, arteriosclerotic lesions, thrombus or tortuosity [1]. Previous endovascular procedures or elongation and kinking of the aorta proximal to the target vessels might increase embolic risk or make access impossible.

The group of Makaloski et al. [3] recently published a paper to describe a retrograde access to antegrade branches with steerable sheaths. We also used this technique over the recent years. The aim of this paper is to describe our experience and differences compared to Kölbel’s technique.

## Technique

We used this technique in 12 patients (33 branches, mean age 72, nine men) to repair complex abdominal and thoraco-abdominal aortic aneurysms (three to four antegrade branches per patient). All patients were operated in a hybrid operating room (Siemens Artis Zee Floor). We performed surgery under general anesthesia with a spinal fluid drainage in eight patients and near-infrared spectroscopy – monitoring in all patients (Medtronic INVOS, frontal, thoracic and lumbar). The patients were systemically heparinized with an activated clotting time (ACT)>250 s during surgery.

**Figure 1:**
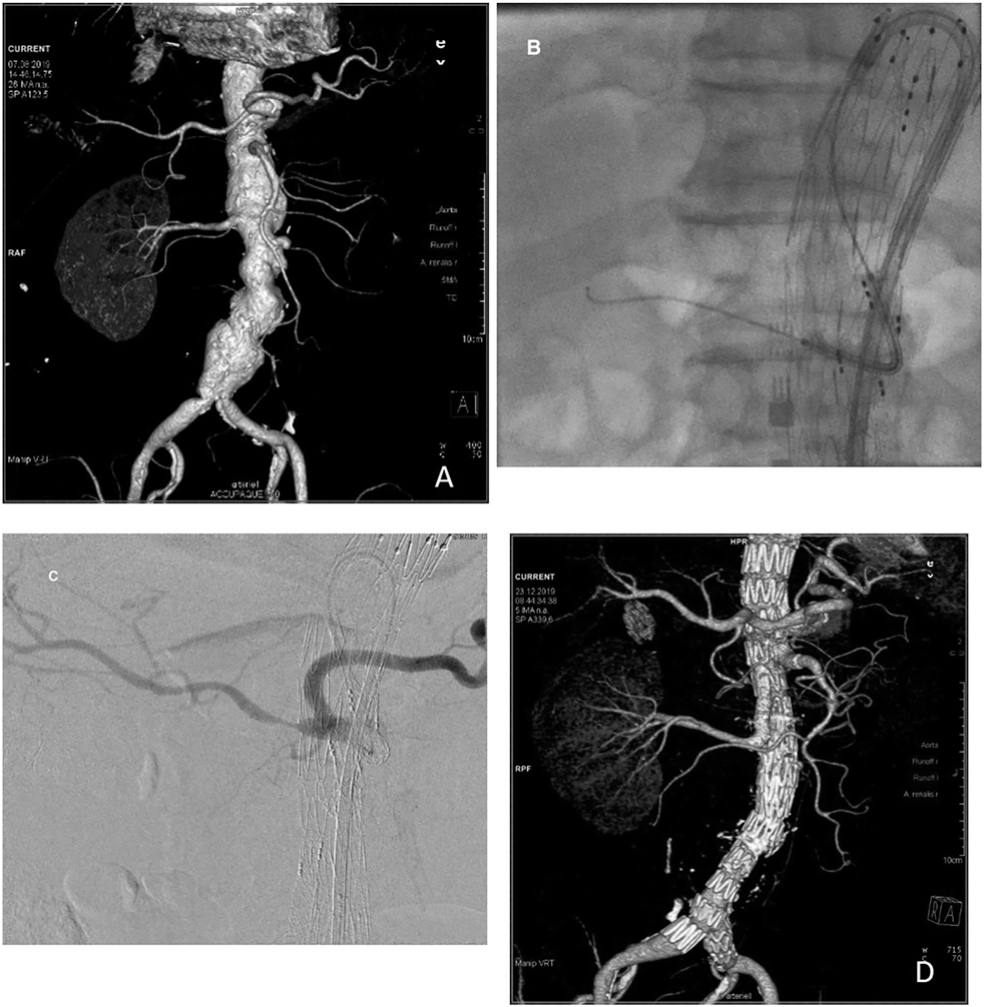
A seventy-three-year-old man with a 58-mm type 4 thoraco-abdominal aneurysm. (A) He underwent nephrectomy after a car accident 20 years ago. In a 2-stage procedure, a custom-made 3-branch endograft (Cook Zenith) was implanted. (B and C) With the steerable sheath in place stabilized by the through-and-through wire, precise placement of the bridging stent (Gore Viabahn BX 9 × 59 mm) is done. (D) The postoperative control shows complete exclusion of the aneurysm.

**Figure 2:**
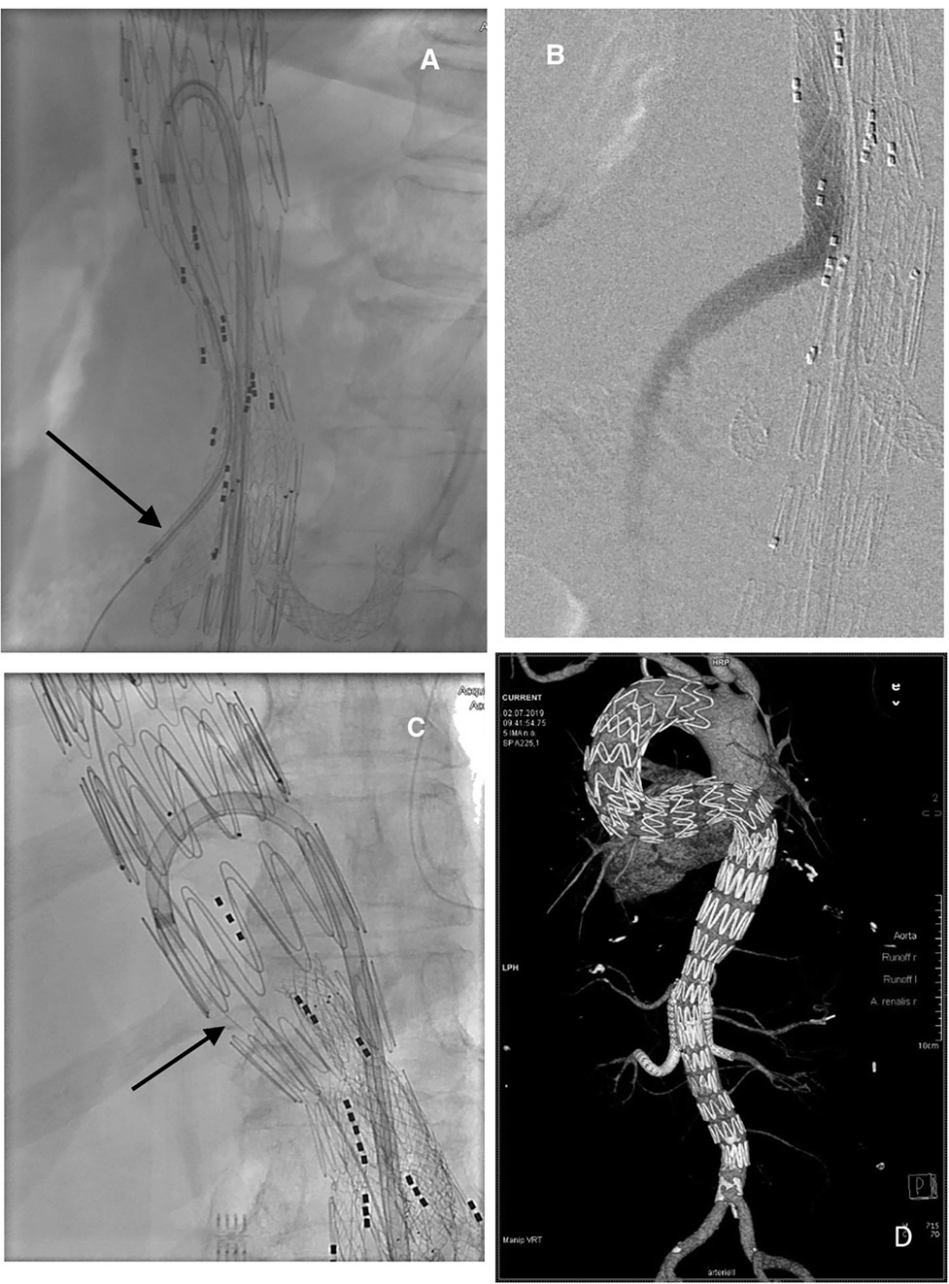
A seventy-year-old woman with a 68-mm type 2 thoraco-abdominal aortic aneurysm and massive kinking of the descending thoracic aorta. In a staged procedure, three thoracic endografts and an off-the-shelf 4-branch endograft (Cook Zenith T-Branch) were implanted. (A) As demonstrated on the superior mesenteric artery (SMA)-branch, the curved sheath with the through-and-through wire in place allows for precise placement of the bridging stent graft (Gore Viabahn BX 9 × 59 mm) without the need of intubating the branch with the sheath. (B) Control angiography is done via a 4 F catheter. (C) The 14″ through-and-through wire is demonstrated by the black arrow. (D) The postoperative control demonstrates the massive kinking of the descending thoracic aorta. An antegrade approach to the branches would have been challenging.

We used a steerable 8.5 F sheath with 65 cm working length (17 mm curve, standard shaft, Destino Twist, Oscor Inc., Palm Harbor, FL 34683, USA) via a femoral access. In cases with tortuous or calcified iliac arteries, an additional 14 F sheath can be helpful to facilitate pushability and steerability of the 8.5 F sheath. After the sheath was placed 5–6 cm proximal to the branches, a 0.014″ wire (Stabilizer 260 cm, Cordis, 6340 Baar, CH) was advanced from the contralateral femoral access and pulled into the sheath with a snare (EN Snare, Merit Medical, UT 84095, US) resulting in a through-and-through wire situation. Now, the tip of the steerable sheath was deflected and positioned 2–3 cm proximal to the targeted branch. Parallel to the stabilizer wire, a 4 F catheter (e.g., vertebralis, 100 cm) was advanced over a hydrophilic 0.035″ guidewire. After catheterization of the branch and the target vessel ostium, the wire was exchanged for a 260-cm Rosen wire (Cook Medical Europe, Bjaeverskov, DK), and a balloon-expandable bridging stent (Gore Viabahn BX, AZ86001, US) was placed and deployed. During this maneuver, the sheath was held in position with slight traction on both ends of the through-and-through wire. Additional smaller sheaths are not used. We avoided contact between the sheath and the ostium of the branch to prevent displacement of the branched main graft. We usually started with the most distal branch and went to the next proximal one to avoid interactions between the proximal part of the bridging stent in place and the one on the balloon. We did not observe problems caused by obstruction of branches by the steerable sheath during the procedure. After completion, the through-and-through wire was removed; the steerable sheath was straightened and then pulled back (Figures 1 and 2).

All 33 branches of 12 patients were successfully completed with this technique. There was a superior stability of the curved tip of the steerable sheath, especially while advancing the bridging stent.

## Discussion

Target vessel catheterization is one of the most difficult steps in complex endovascular procedures. In branched stent grafts, downward-oriented branches usually require an upper extremity access via brachial, axillary or subclavian artery. Even with stabilizing the access sheath with a through-and-through wire and maintaining ACT>250 s, there is a significant risk of cerebral embolization and stroke [1], [2].

Steerable sheaths have been successfully used as a secondary option after failed standard cannulation in fenestrated and branched endovascular repair [4]. Even “self-made” steerable sheaths have been published and can be helpful in bailout situations when no standard steerable sheath is available [5].

Makaloski et al. [3] were the first to report 4 cases in which they used a retrograde access with a steerable sheath to complete antegrade branches. They emphasized stability, pushability and torquebility of the steerable sheath. We also used this approach but found that during wire exchange, and especially during advancement of the bridging stent over the curved part, the sheath stability is partially lost by the sheath giving up its 180° configuration. As a result, we lost the wire from the branch or could not further advance the bridging stent in several target vessels. With the modification of a through-and-through wire, the 180° configuration and the position of the sheath is completely stable during wire exchange and does not change when the bridging stent is advanced. As a result, the number of cannulation attempts, fluoroscopy time and procedure time are reduced. In comparison to an upper extremity access, an 8.5 F steerable sheath is used instead of a 12 F and an 8 F flexor sheath with similar costs.

There might be a potential risk of obstruction of branch ostia by the sheath during the procedure but we did not observe that.

## Conclusion

The modified retrograde femoral access to antegrade branches using a steerable sheath and a through-and-through wire seems to be safe and effective in complex endovascular aortic repair. Retrograde femoral access may help to reduce the potential risk of periprocedural stroke by making an upper extremity approach unnecessary.
