# Supplementary material for: Improved stability of steerable sheath access by femoro-femoral crossover wire in branched stent graft repair of complex thoraco-abdominal aortic aneurysms
Source: Innov Surg Sci. 2020 Sep 7;5(1-2):63–5. doi: 10.1515/iss-2020-0006 (PMC7798303; doi:10.1515/iss-2020-0006)
Supplement: Supplementary file 1 [file iss-5-20200006-s001.pdf]

Reviewer Assessment

Ingolf Töpel et al.: Improved stability of steerable sheath access by femoro-femoral crossover wire in branched stentgraft repair of complex thoraco-abdominal aortic aneurysms

Reviewers' Comments to Original Submission

Reviewer 1: anonymous

Date received: 29-Feb-2020
Reviewer recommendation: **Accept in present form**
Reviewer overall scoring: **High**

Assessment Form scores: 5 = High/Yes; 3 = Medium/Adequate; 1 = Low

Is the subject area appropriate for the journal					
Does the title clearly reflect the paper's content?	5				
Does the abstract clearly reflect the paper's content	5				
Do the keywords clearly reflect the paper's content?	5				
Does the introduction present the problem clearly?	5				
Are the results/ conclusions justified?		4			
How comprehensive and up-to-date is the subject matter presented?		4			
How adequate is the data presentation?	5				
Are units and terminology used correctly?	5				
Is the number of cases adequate?	5				
Are the experimental methods/ clinical studies adequate?	5				
Is the length appropriate in relation to the content?		4			
Does the reader get new insights from the article?		4			
Please rate the practical significance.		4			
Please rate the accuracy of methods.	5	4			
Please rate the statistical evaluation and quality control.		4			
Please rate the appropriateness of the figures and tables.	5				
Please rate the appropriateness of the references.	5				
Please evaluate the writing style and use of language.	5				
Please judge the overall scientific quality of the manuscript.		4			
Are the methods used worthy of reproduction in greater deal?	yes				

Comments to author: This paper puts a focus on an important innovation during complex endovascular aortic repair. The paper is well written, not too lengthy and is very didactic. The reader can easily understand, what has been done here. The figures are helpful, additionally.

Reviewer 2: anonymous

Date received: 27-Jun-2020
Reviewer recommendation: **Accept in present form**
Reviewer overall scoring: **Medium**

Assessment Form scores: 5 = High/Yes; 3 = Medium/Adequate; 1 = Low

Is the subject area appropriate for the journal					
Does the title clearly reflect the paper's content?	5				
Does the abstract clearly reflect the paper's content	5				
Do the keywords clearly reflect the paper's content?	5				
Does the introduction present the problem clearly?	5				
Are the results/ conclusions justified?			3		
How comprehensive and up-to-date is the subject matter presented?			3		
How adequate is the data presentation?					1
Are units and terminology used correctly?	5				
Is the number of cases adequate?					1
Are the experimental methods/ clinical studies adequate?	5				
Is the length appropriate in relation to the content?	5				
Does the reader get new insights from the article?			3		
Please rate the practical significance.			3		
Please rate the accuracy of methods.			3		
Please rate the statistical evaluation and quality control.			3		
Please rate the appropriateness of the figures and tables.			3		
Please rate the appropriateness of the references.	5				
Please evaluate the writing style and use of language.	5				
Please judge the overall scientific quality of the manuscript.			3		
Are the methods used worthy of reproduction in greater deal?	yes				

Comments to author: The most important thing is missing: a detailed description of the 12 cases where this technique was used. What the advantages are in real life compared to the technology described by Kölbel is open. The authors write: "There was a superior stability of the curved tip of the steerable sheath especially while advancing the bridging stent."
What did this actually mean clinically or for the procedure time or radiation time?
